# Development of a PCR Assay Targeting the *iboH* Gene to Assess the Distribution of Ibotenic Acid/Muscimol Biosynthesis Potential in the Genus *Amanita*

**DOI:** 10.3390/jof12090691

**Published:** 2026-09-16

**Authors:** Zheng-Mi He, Jia-Xi Li

**Affiliations:** Hunan Research Center of the Basic Discipline Developmental Biology, College of Life Sciences, Hunan Normal University, Changsha 410081, China; jiaxili@hunnu.edu.cn

**Keywords:** ibotenic acid, muscimol, molecular detection, mushroom poisoning

## Abstract

Ibotenic acid and muscimol, synthesized by the ibotenic acid biosynthetic gene cluster (*ibo* BGC) in *Amanita* species, are two of the primary neurotoxins responsible for mushroom poisoning incidents worldwide. However, the molecular detection methods for these toxins remain underdeveloped. In this study, we designed a pair of universal PCR primers targeting *iboH*, a hydroxylase-encoding gene within the *ibo* BGC, and developed a PCR assay with a detection limit of 10 pg of genomic DNA per reaction. Using the assay on 53 *Amanita* species, 21 out of the 23 species tested from section *Amanita* were positive, whereas all 30 species from sects. *Amidella* (1), *Caesareae* (3), *Phalloideae* (6), *Roanokenses* (8), *Strobiliformes* (1), *Vaginatae* (4), and *Validae* (7) tested negative. We then integrated these PCR signals into our global phylogenetic analysis of *Amanita* and found that *iboH* is restricted to a large clade within sect. *Amanita. Amanita sinensis*, commonly considered an edible species in China, was detected carrying *iboH*, indicating its potential to produce ibotenic acid/muscimol and pose associated health risks. In conclusion, this study establishes an effective universal PCR assay for detecting ibotenic acid/muscimol-producing *Amanita* species and offer a reference for toxigenic mushroom recognition and food safety.

## 1. Introduction

*Amanita* (Amanitaceae, Agaricales) is a cosmopolitan genus comprising 11 sections and over 650 species [1,2]. It is well-known for its edible species but even more notorious for its poisonous ones [3]. Among them, section *Amanita*, with 112 accepted species reported [2,4,5,6,7], is one of the major groups responsible for neurotoxicity in mushroom poisoning worldwide [8]. This section is characterized by an agaricoid basidioma that has a pileus bearing a striate margin and persistent volval remnants on its surface, a stipe with basal bulb, and inamyloid basidiospores [9,10]. People may easily mistake some poisonous species of this section for other edible *Amanita* mushrooms like *A. caesarea*, *A. hemibapha*, and *A. rubescens*. Indeed, species of this section have caused numerous poisoning incidents, even resulting in fatalities [11,12]. In Europe and North America, *A. muscaria* and *A. pantherina* are the species most frequently reported [13,14], whereas in China, the main species involved include *A. melleiceps*, *A. rufoferruginea*, *A. subglobosa*, and *A. sychnopyramis* [15]. From 2019 to 2024, a total of 147 poisoning incidents involving 492 patients related to these species were documented in China [16,17,18,19,20,21], which highlights the urgent need for rapid and reliable identification and risk assessment methods.

The primary neurotoxins in sect. *Amanita* are ibotenic acid (IBO) and muscimol (MUS), two isoxazoles that are structurally analogous to glutamic acid and gamma-aminobutyric acid (GABA) [22]. Both toxins can stimulate N-methyl-D-aspartate (NMDA) and GABA receptors, thereby inducing central nervous system symptoms, such as dizziness, somnolence, delirium, dysphoria, and hallucinations [23]. These compounds were initially isolated from *A. muscaria* [24,25] and have since been detected in 15 other species of this section [6,15,26,27,28,29,30,31]: *A. altipes*, *A. concentrica*, *A. cothurnata*, *A. flavopantherina*, *A. gemmata*, *A. griseopantherina*, *A. ibotengutake*, *A. pantherina*, *A. parvisychnopyramis*, *A. pseudopantherina*, *A. pseudosychnopyramis*, *A. regalis*, *A. rubrovolvata*, *A. subglobosa*, and *A. sychnopyramis*.

The biosynthesis pathways of IBO and MUS were recently elucidated by Obermaier and Müller [32]. Their findings revealed that the synthesis of these compounds is derived from the amino acids glutamine or glutamate and involves seven core enzymes encoded by a biosynthetic gene cluster (BGC): IboA (adenylating), IboC (cytochrome P450), IboD (decarboxylase), IboF (flavin-dependent monooxygenase), IboG1 and G2 (two pyridoxal phosphate-dependent enzymes), and IboH (hydroxylase). Su et al. [33] identified this *ibo* BGC in 21 Chinese species that form a major clade within sect. *Amanita*, suggesting a monophyletic distribution of the BGC and a widespread genetic capacity for IBO/MUS production within this section. However, whether the *ibo* BGC is present in other sections of *Amanita* has not been sufficiently investigated through extensive sampling.

Currently, liquid chromatography–mass spectrometry (LC-MS) is the conventional method for detecting IBO and MUS [29,34]. This method can be applied in the detection of mushroom or human urine samples for the investigation of food poisoning cases [35,36]. However, it requires expensive instrumentation, chemical standards, and professional personnel, thus impeding its application in large-scale or routine screenings. In comparison, PCR-based molecular assays offer distinct advantages: (1) PCR instruments and reagents are far more affordable, and the workflow is simpler, which is particularly advantageous in resource-limited settings; (2) PCR allows multiplexed analysis and typically 96 samples to be processed in a single run, whereas LC-MS processes samples individually, which drastically reduces throughput and becomes time-consuming for large-scale analyses. However, to date, there has been no simple and rapid molecular assay available to infer the presence of these toxins based on their biosynthetic genes.

In our previous work [33], we retrieved the *ibo* BGC genes from 21 *Amanita* species, including 21 *iboA*, 19 *iboC*, 20 *iboD*, 19 *iboF*, 19 *iboG1*, 18 *iboG2*, and 18 *iboH*, thereby laying the foundation for the design of specific primers. In this study, we selected the 18 *iboH* sequences obtained from our previous work [33] as target templates for primer design, because that *iboH* is a 620-bp, intron-free, and highly conserved gene indispensable for glutamate hydroxylation in the IBO/MUS biosynthesis. Based on these *iboH* sequences, we designed specific primers and developed a universal PCR-based method targeting *iboH*. Applying this method to 53 *Amanita* species from eight sections, we investigated the occurrence and distribution of *iboH* across the genus *Amanita*. This PCR-based method provides a simple, rapid, and cost-effective alternative to LC-MS, with applications in clinical diagnostics and prevention, food safety monitoring, and epidemiological surveillance of mushroom poisoning.

## 2. Materials and Methods

### 2.1. Fungal Specimens and Identification

**Figure 1:**
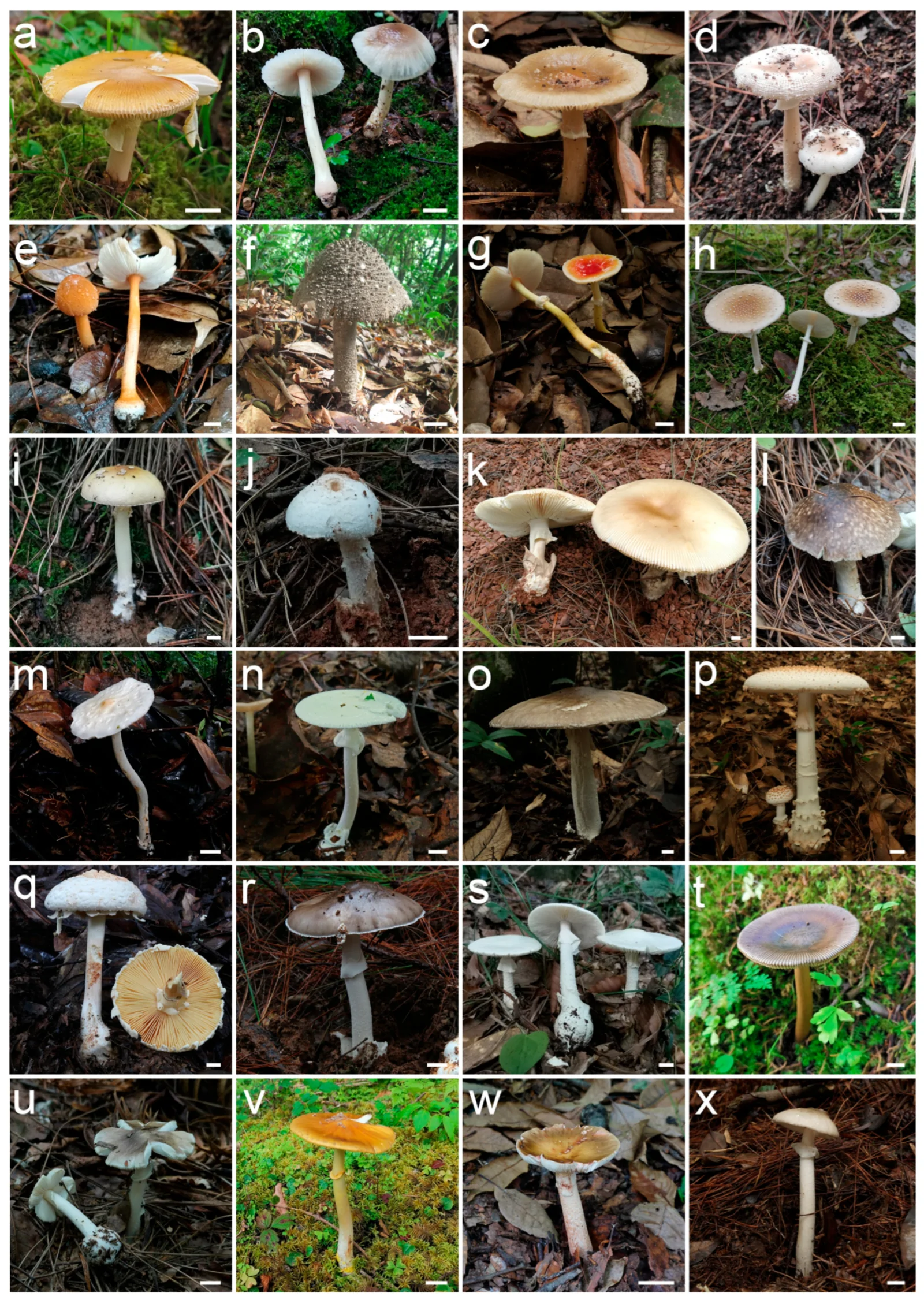
Basidiomata of representative *Amanita* species from different sections. Section *Amanita*: (**a**) *A. altipes* (MHHNU 33050); (**b**) *A. farinosa* (MHHNU 32693); (**c**) *A. parvipantherina* (MHHNU 32942); (**d**) *A. pseudosychnopyramis* (MHHNU 32762); (**e**) *A. rufoferruginea* (MHHNU 31100); (**f**) *A. sinensis* (MHHNU 8585); (**g**) *A. subfrostiana* (MHHNU 32955); (**h**) *A. subglobosa* (MHHNU 32538); (**i**) *A. subparvipantherina* (MHHNU 32226); sect. *Amidella*: (**j**) *A. rufobrunnescens* (MHHNU 33470); sect. *Caesareae*: (**k**) *A. princeps* (MHHNU 31274); (**l**) *A. yuaniana* (MHHNU 34553); sect. *Phalloideae*: (**m**) *A. exitialis* (MHHNU 20088); (**n**) *A. pallidorosea* (MHHNU 10523); sect. *Roanokenses*: (**o**) *A. caojizong* (MHHNU 32855); (**p**) *A. eijii* (MHHNU 10639); (**q**) *A. gymnopus* (MHHNU 33197); (**r**) *A. manginiana* (MHHNU 34165); (**s**) *A. oberwinklerana* (MHHNU 34779); sect. *Vaginatae*: (**t**) *A. umbrinolutea* (MHHNU 34330); sect. *Validae*: (**u**) *A. brunneolimbata* (MHHNU 33436); (**v**) *A. flavoconia* (MHHNU 34414); (**w**) *A. rubescens* (MHHNU 34775); (**x**) *A. submaculata* (MHHNU 32842). Scale bars = 1 cm.

A total of 53 *Amanita* specimens collected from China were used in this study. Detailed information for each specimen is provided in Table 1. Fresh basidiomata (Figure 1) were photographed in situ using a Sony ILCE-7 camera (Sony Inc., Tokyo, Japan). Specimens were dried at 45 °C using a fruit dehydrator (KD728, Kakool, Guangzhou, China) and accessioned at the Mycological Herbarium of Hunan Normal University (MHHNU, Changsha, China). Specimens were identified based on morphological characters using the monographs of Yang et al. [9,10] and Cui et al. [1]. Identifications were further verified by DNA barcode sequencing and phylogenetic analysis as described below.

### 2.2. DNA Extraction, Primer Design, PCR Amplification, and Sequencing

**Figure 2:**
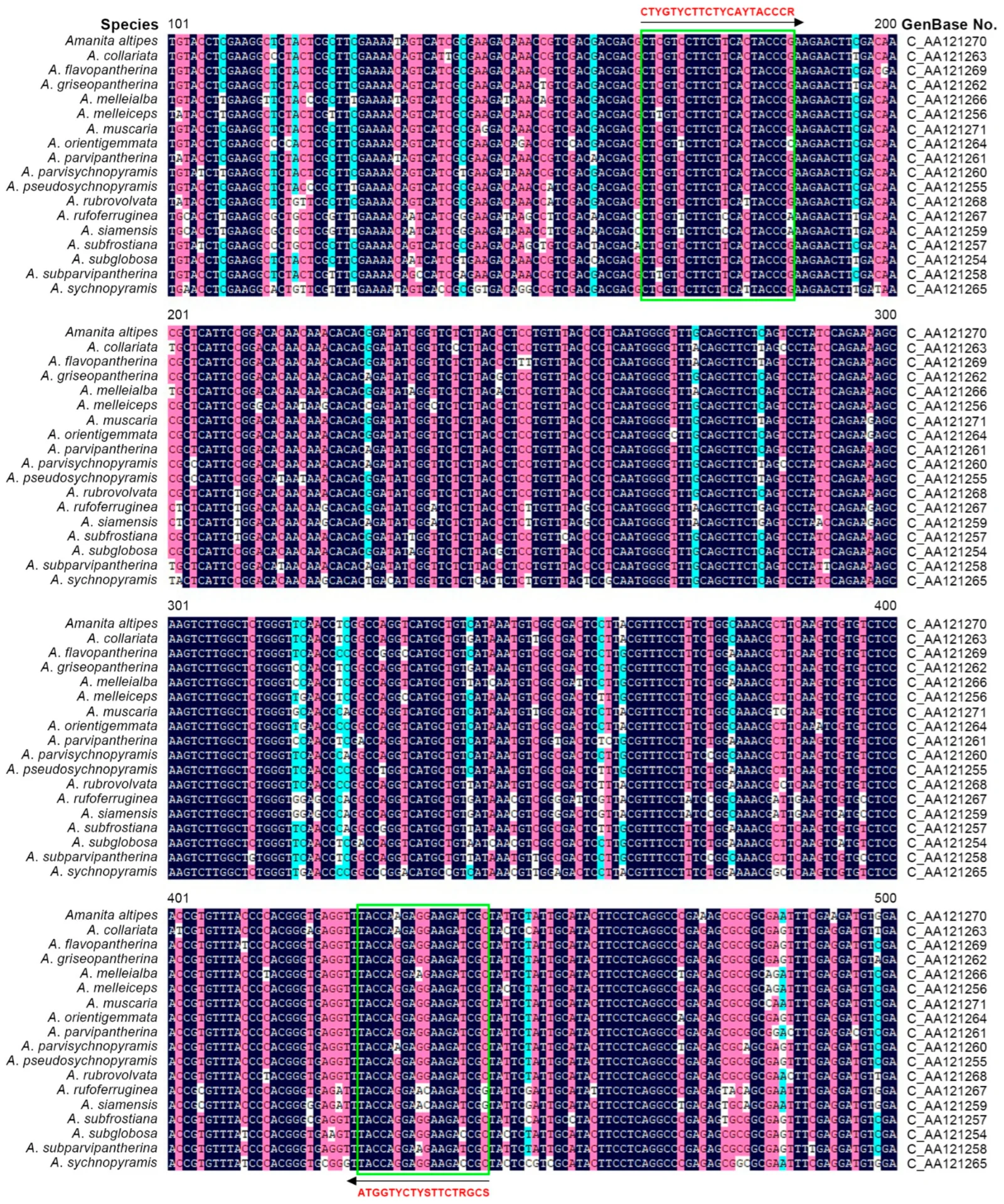
Multiple sequence alignment of *iboH* genes from 18 *Amanita* species, showing the conserved region (nucleotide positions 101–500) used for primer design. Binding sites of the forward and reverse primers are indicated by arrows. Sequences were downloaded from GenBase [93].

Total genomic DNA was extracted using the Fungal DNA Mini Kit (Omega Bio-Tek, Norcross, GA, USA) according to the manufacturer’s instructions. Universal PCR primers targeting *iboH* were designed based on a sequence alignment of *iboH* genes from 18 *Amanita* species reported by Su et al. [33] (Figure 2). The designed primer pair was IboH-F (5′-CTYGTYCTTCTYCAYTACCCR-3′, 21 bp) and IboH-R (5′-SCGRTCTTSYTCYTGGTA-3′, 18 bp), yielding a predicted amplification product of 279 bp. The specificity of the primers was further verified using Primer-BLAST v2.5.0 [82]. The internal transcribed spacer (ITS) was amplified using the primer pair ITS5/ITS4 [83], and the nuclear ribosomal large subunit (LSU) was amplified using LR0R/LR5 [84]. These primers were synthesized by Tsingke Biotechnology Co., Ltd. (Beijing, China).

The PCR mixture was prepared in a total volume of 25 μL, consisting of 1× Hieff^®^ PCR Master Mix (Yeasen Biotechnology, Shanghai, China; providing 1× PCR buffer, 1.5 mM MgCl_2_, 0.2 mM dNTPs, and 1.25 U of *Taq* polymerase), 0.4 μM forward primer, 0.4 μM reverse primer, and 1 μL of DNA template. PCR was performed in an ABI 2720 thermal cycler (Applied Biosystems, Foster City, CA, USA). The thermal cycling program consisted of an initial denaturation at 94 °C for 5 min, followed by 35 cycles of denaturation at 94 °C for 30 s, annealing at 52 °C (LSU), 54 °C (ITS) or 56 °C (*iboH*) for 30 s, and extension at 72 °C for 30 s (ITS and *iboH*) or 60 s (LSU), with a final extension at 72 °C for 10 min.

PCR products were examined by electrophoresis on a 2% agarose gel in 1× TAE buffer (Solarbio, Beijing, China) at 110 V for 15 to 20 min. A DL2000 DNA marker (BioRun, Wuhan, China) was used as the molecular weight standard. Gels were photographed under a JP-2880 gel documentation system (Jiapeng, Shanghai, China). The successful amplicons were sent to Sangon Biotech Co., Ltd. (Shanghai, China) for sequencing.

### 2.3. Specificity and Sensitivity of PCR Assay Targeting iboH

To evaluate the specificity of the specific PCR primers IboH-F/IboH-R designed above, genomic DNA from 53 *Amanita* species representing eight sections of the genus (Table 1) was tested. For sect. *Amanita*, 18 species had been confirmed to carry *iboH* and two species (*A. farinosa* and *A. subparcivolvata*) had been confirmed to lack *iboH* by Su et al. [33]. Three sect. *Amanita* species (*A. flavomelleiceps*, *A. sinensis*, and *A. pseudopantherina*) had not been previously confirmed. The status of *iboH* in the remaining 30 tested species from seven other sections were unknown. For each PCR run, ddH_2_O was used as the blank control in place of DNA template, and all samples were tested in at least three independent replicates to ensure reproducibility.

To determine the limit of detection (LOD), the PCR tests were performed using serial 10-fold dilutions of the genomic DNA from *A. altipes* (MHHNU 33050) ranging from 1 μg to 1 pg.

### 2.4. Identification of iboH Genes

Chromatograms from bidirectional sequencing of *iboH* amplicons were visualized using BioEdit v7.2.5 [85] to check peak quality and to confirm that these nucleotide sequences (excluding primers) were 240 bp in length. The trimmed nucleotide sequences were translated into amino acid sequences and aligned with the IboH sequence of *A. muscaria* Koide BX008 (KIL56739) using DNAMAN v7.212 (Lynnon Biosoft, Vaudreuil-Dorion, QC, Canada) for identification.

### 2.5. Phylogenetic Analysis

To achieve a comprehensive and high-coverage phylogenetic analysis, sequences of 112 representative *Amanita* species encompassing all 11 sections of the genus were included (Table 1). Among these, sequences of 53 species were obtained from our Chinese specimens, while sequences of the remaining 59 species, originating from Africa, Europe, North America, Oceania, and other regions of Asia, were downloaded from GenBank (https://ncbi.nlm.nih.gov; accessed on 7 March 2026). ITS and LSU sequences (Table 1) were aligned using MAFFT v7.511 [86]. Ambiguously aligned regions were trimmed using Gblocks v0.91b [87]. The two loci were concatenated using SequenceMatrix 1.7.8 [88]. The generated dataset for *Amanita* comprised 1414 positions from 163 samples (Supplementary Material S1).

According to the AIC criterion in MrModeltest v2.4 [89], GTR + I + G was estimated as the best-fit model for the ITS and LSU. Bayesian inference (BI) analysis was performed in MrBayes v3.2.7 [90] using the selected model, with two simultaneous runs, four Markov Chain Monte Carlo (MCMC) chains, and sampling every 1000 generations over a total of 5,000,000 generations, until the average standard deviation of split frequencies fell below 0.01. The first 25% generations were discarded as burn-in, and convergence was visually assessed using the trace function in Tracer v1.7.2 [91]. Maximum likelihood (ML) analysis with 1000 bootstrap replicates was computed in RAxML v8.0.20 [92] using the GTR + G + I model.

## 3. Results

### 3.1. PCR Assay Targeting iboH and Sequence Identification

**Figure 3:**
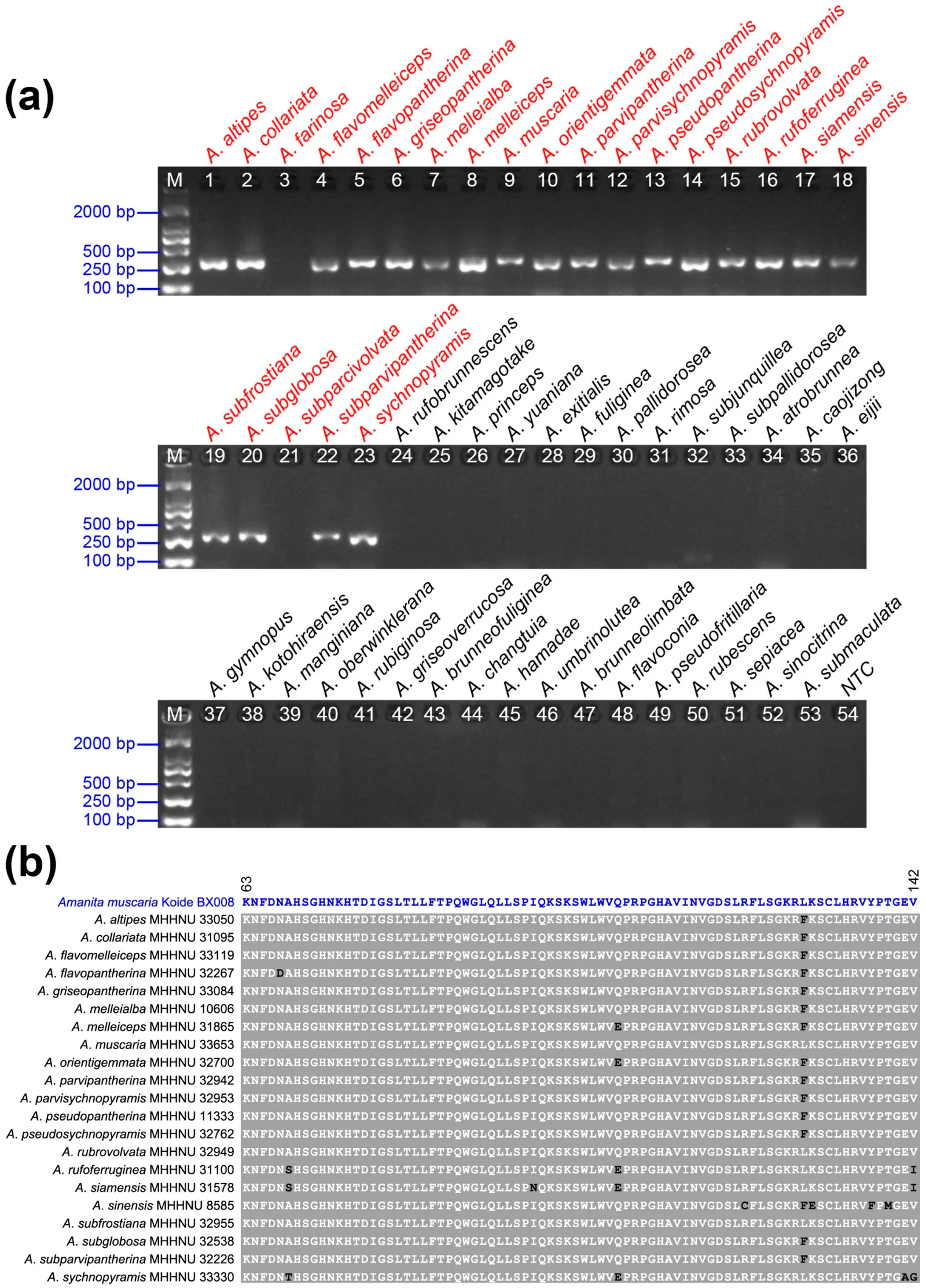
PCR detection of *iboH* in *Amanita* species. (**a**) Agarose gel showing amplification results from representative species; (**b**) alignment of the obtained sequences with the reference IboH sequence from *A. muscaria* Koide BX008 (GenBank: KIL56739) over the region spanning positions 63–142. M, DL 2000 DNA marker; lanes 1–23, sect. *Amanita* (names in red); lane 24, sect. *Amidella*; lane 25–27, sect. *Caesareae*; lane 28–33, sect. *Phalloideae*; lane 29–41, sect. *Roanokenses*; lane 42, sect. *Strobiliformes*; lane 43–46, sect. *Vaginatae*; lane 47–53, sect. *Roanokenses*; lane 54: no template control (NTC).

To detect the occurrence of the *iboH* gene in *Amanita*, DNA samples extracted from 53 *Amanita* species belonging to eight sections were tested. The electrophoretic results are shown in Table 1 and Figure 3a. Overall, 21 species tested positive, yielding bands slightly larger than 250 bp (expected size: 271 bp), while the remaining 32 species and the no temple control tested negative. At the section level, positive reactions occurred only in sect. *Amanita* [21/23], whereas negative reactions occurred in all other sections: *Amidella* [0/1], *Caesareae* [0/3], *Phalloideae* [0/6], *Roanokenses* [0/8], *Strobiliformes* [0/1], *Vaginatae* [0/4], and *Validae* [0/7].

Sequencing results (Table 1) further showed that all positive amplicons were 240 bp in length after removing the primer regions (21 bp for IboH-F and 18 bp for IboH-R), which was consistent with the expected size. The deduced amino acid sequences aligned well with the template IboH sequence from *A. muscaria* Koide BX008 (GenBank accession no. KIL56739) [32], showing 0–5 amino acid differences (Figure 3b). These results validate the identity of the positive amplicons and confirmed the specificity of the designed primers.

**Figure 4:**
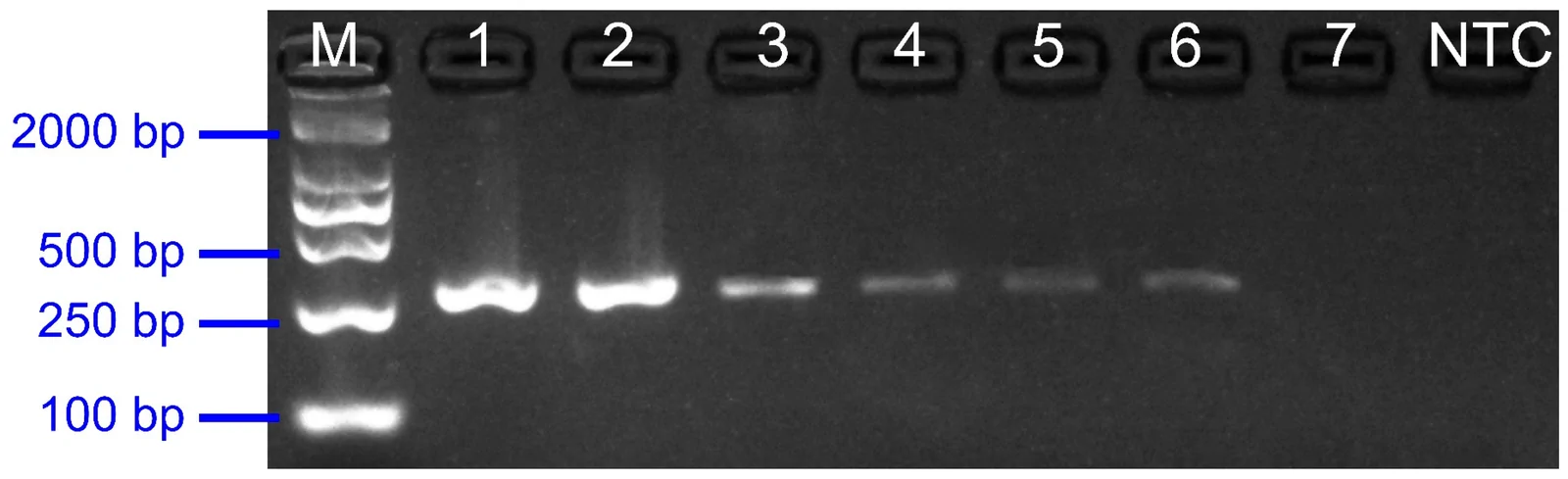
Sensitivity of PCR assay targeting *iboH*. M, DL 2000 DNA marker; NTC, no template control. A ten-fold dilution series of genomic DNA of *A. altipes* was tested: lane 1, 1 μg; lane 2, 100 ng; lane 3, 10 ng, lane 4, 1 ng; lane 5, 100 pg; lane 6, 10 pg; lane 7, 1 pg.

To determine the detection limit, the PCR was performed using a serial 10-fold dilution ranging from 1 μg to 1 pg of DNA template of *A. altipes*. The electrophoresis result (Figure 4) showed that the detection limit of the PCR assay was 10 pg of genomic DNA per reaction.

### 3.2. Phylogenetic Distribution of iboH Within Amanita

**Figure 5:**
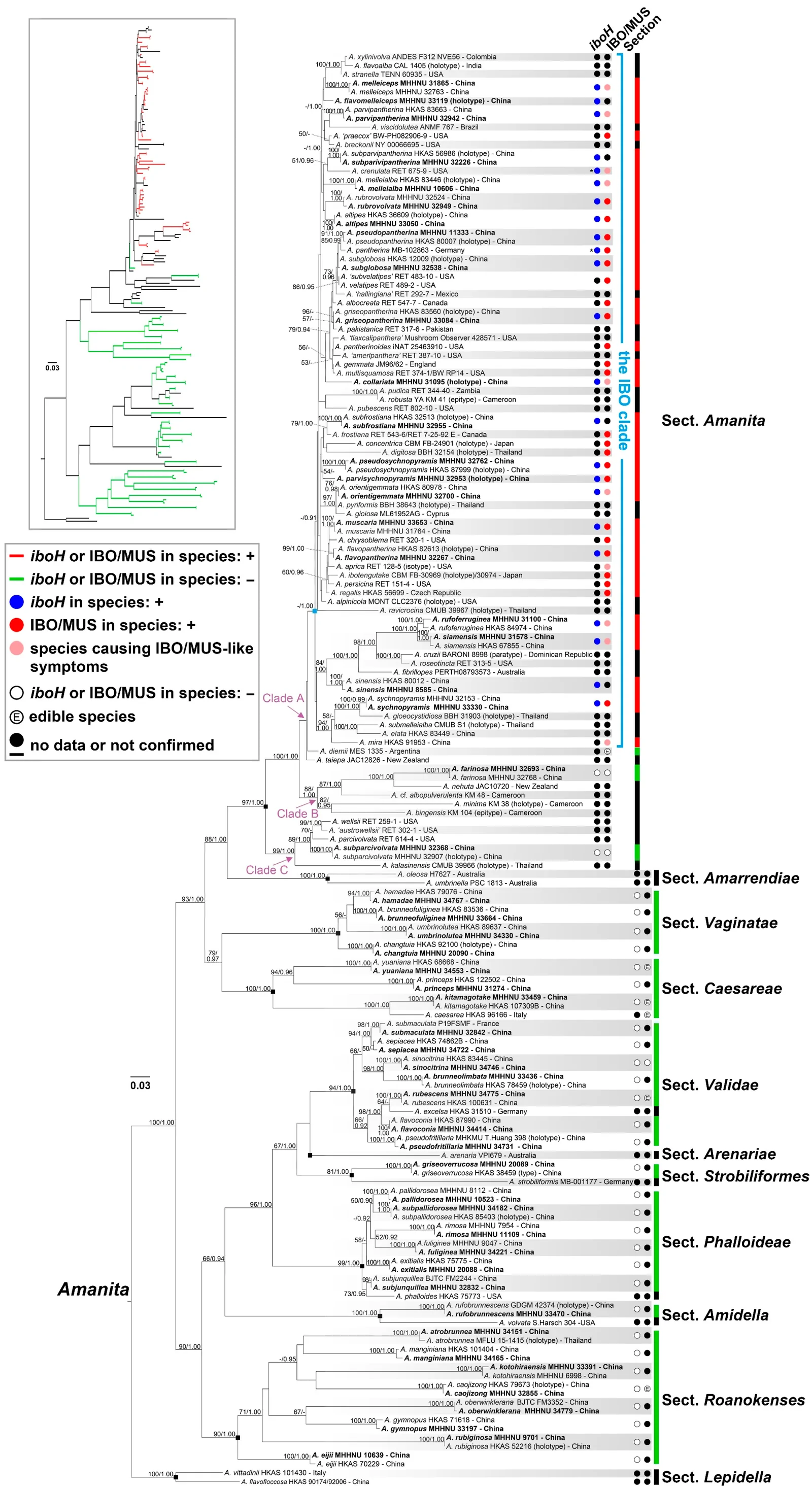
ML analysis of *Amanita* based on concatenated ITS-LSU sequence data. Bootstrap values (BP) ≥ 50% from ML analysis and Bayesian posterior probabilities (PP) ≥ 0.90 from BI analysis are shown at nodes. Geographic origins of specimens are presented, with type specimens in parentheses and Chinese specimens from this study in bold. Clades A, B, and C of Su et al. [33] are indicated by arrows. Red and green lines indicate the presence and absence of *iboH*/IBO/MUS, respectively. Blue, red, and pink circles to the right of the tree indicate species with genomic, chemical, and poisoning evidence for IBO/MUS, respectively, while white circles indicate species without *iboH*/IBO/MUS. “E” indicates edible species, while black lines and circles indicate lack of data or unconfirmed. For the *iboH* gene, data sourced from Obermaier and Müller [32] are marked with asterisks (*), while all other *iboH* data were generated in the present study. IBO/MUS detection data and poisoning case records were compiled from previously published studies, with the corresponding citations provided in Table 1.

Our global phylogenetic analysis of *Amanita* (Figure 5) recovered 112 species. Among them, our Chinese specimens were confirmed to represent 53 known species. The analysis also recovered 11 sections: *Amanita* (97% BP, 1.00 PP), *Amarrendiae* (100% BP, 1.00 PP), *Amidella* (100% BP, 1.00 PP), *Amidella*, *Caesareae* (100% BP, 1.00 PP), *Phalloideae* (100% BP, 1.00 PP), *Roanokenses* (90% BP, 1.00 PP), *Strobiliformes* (81% BP, 1.00 PP), *Vaginatae* (100% BP, 1.00 PP), and *Validae* (94% BP, 1.00 PP). The section *Amanita* was further divided into three major clades and two species lineages: the IBO clade (42% BP, 1.00 PP), Clade B (88% BP, 1.00 PP), Clade C (99% BP, 1.00 PP), and the *A. diemii* and *A. taiepa* lineages.

As shown in Figure 5, all 21 *iboH*-positive species, together with the IBO/MUS-containing species and those previously reported to cause human poisoning with symptoms consistent with IBO/MUS exposure, fell within the IBO clade of sect. *Amanita*. In contrast, all 32 *iboH*-negative species, along with species reported to lack IBO/MUS and edible species were all recovered outside this clade.

## 4. Discussion

### 4.1. ibo BGC Distribution Within Amanita and Delimitation of the IBO Clade

In our previous work [33], we identified the *ibo* BGC in 20 known *Amanita* species by shallow whole-genome sequencing, but failed to detect *iboH* in *A. flavomelleiceps* and *A. pseudopantherina*. In the present study, we revisited these species using PCR and successfully confirmed the presence of *iboH* in all 20 species. We additionally detected the gene in *A. sinensis*. Given that *iboH* is a core gene within the *ibo* BGC [32], the presence of this gene implies the existence of the complete BGC in these species. The BGC was also found in *A. crenulata* and *A. pantherina* [32]. Another 11 species, viz. *A. albocreata* [37], *A. concentrica* [15], *A. digitosa* [50], *A. frostiana* [54], *A. gemmata* [26], *A. ibotengutake* [28], *A. multisquamosa* [37], *A. pantherinoides* [59], *A. persicina* [37], *A. praecox* [37], *A. regalis* [31], and *A. velatipes* [37], have been reported to contain IBO/MUS, and two additional species, *A. aprica* [40] and *A. mira* [57], have been implicated in poisonings with symptoms consistent with IBO/MUS exposure. These observations suggest that the *ibo* BGC is present in all 36 species listed above. Interestingly, all these species fall into a large monophyletic clade within sect. *Amanita*, viz., the IBO clade (Figure 5).

By contrast, *iboH* was not detected in 32 species, which included representatives from Clades B (1) and C (1) of sect. *Amanita*, as well as from sects. *Amidella* (1), *Caesareae* (3), *Phalloideae* (6), *Roanokenses* (8), *Strobiliformes* (1), *Vaginatae* (4), and *Validae* (7). Two additional species, *A. diemii* [48] and *A. caesarea* [67], have been reported to be edible. Therefore, these 34 species are considered to lack the *ibo* BGC and as expected, all of them fall outside the IBO clade (Figure 5).

Collectively, these findings indicate that the *ibo* BGC is restricted to a large clade within sect. *Amanita*, suggesting it has an independent origin within *Amanita*. By extending our sampling from sect. *Amanita* in Su et al. [33] to the entire genus using a PCR approach, we corroborate the earlier report by Su et al. [33] using genomic sequencing and further demonstrate that this restriction is maintained across the genus.

The IBO clade is defined here as the monophyletic group encompassing all *Amanita* species that (i) harbor *ibo* genes, (ii) have been chemically confirmed to produce IBO/MUS, or (iii) are associated with poisoning cases exhibiting symptoms typical of IBO/MUS intoxication. In the ITS-LSU phylogenetic analysis of Su et al. [33], sect. *Amanita* was resolved into three clades, designated as Clades A–C, of which Clades B and C received strong support, whereas Clade A was only weakly supported. In our ITS-LSU analysis (Figure 5), Clades B and C of Su et al. [33] also received strong support, but Clade A of Su et al. [33] received no statistical support. However, after excluding *A. diemii* and *A. taiepa* from Clade A, the remaining species formed a strongly supported clade with 1.00 PP in BI analyses of Su et al. [33] and the present study. Therefore, Clade A as a whole is unstable, while the IBO clade is more likely corresponds to this well-supported clade. Salazar Vidal [48] reported that *A. diemii* has been consumed by the Mapuche people and rural communities in Chile for over a century, which supports our exclusion of this species from the IBO clade. We therefore recognize this clade as the IBO clade, as shown in Figure 5. A more precise delimitation of this clade will require using more genetic loci in phylogenetic analysis and more comprehensive *ibo* gene detection.

### 4.2. Considerations of Edibility and Toxicity of Amanita sinensis

*Amanita sinensis* is generally recorded as an edible species in the literature [94,95] and monographs [9,10], and is known to be consumed locally in Southern China. However, a recently published monograph on Chinese poisonous mushrooms [96] classifies this species as a gastrointestinal irritant, and reports a case in Yunnan Province in which ingestion led to palpitations and nausea in the patients. In this study, we successfully detected *iboH* in this species and confirmed its identity by sequencing (Figure 3b). This finding indicates that this species is highly likely to harbor the *ibo* BGC for the production of IBO/MUS. Accordingly, we consider that consumption of *A. sinensis* may present potential human health risks.

### 4.3. Specificity and Universality of the iboH-Targeted PCR Primers

The *iboH*-targeted PCR primers show high universality, successfully amplifying the target gene from 20 *Amanita* species previously confirmed to possess the *ibo* BGC [33], with no amplification from *A. farinosa* and *A. subparcivolvata*. These two species were confirmed to lack all genes of the *ibo* BGC, including *iboH*, by genome sequencing conducted by Su et al. [33], which is consistent with our PCR results. Despite interspecific *iboH* differences, all tested *iboH*-containing species were consistently detected (Figure 3a). The specificity was further validated by Sanger sequencing, which confirmed the identity of all products as *iboH* (Figure 3b). Notably, a very faint band of approximately 100 bp was observed for *A. subjunquella* (lane 32, Figure 3a), which represents a non-specific amplification. However, this product is clearly distinct from the expected 271 bp *iboH* amplicon and given its low intensity, it did not interfere with the visual interpretation or downstream sequencing validation.

### 4.4. Comparison Between PCR and LC-MS for Detection of IBO/MUS

LC-MS detects the toxins IBO and MUS themselves, providing direct identification and quantification of the actual chemical compounds present in samples. It is applicable to diverse matrices including mushrooms [97], human urine [98], serum [99], and gastric contents [100], making it the gold standard for clinical diagnosis and forensic investigation of poisoning cases. However, the technique requires expensive instrumentation and chemically standards, well-trained personnel, and complicated sample preparation and experiment operation. Additionally, IBO and MUS are chemically unstable and prone to degradation [15,101], which may lead to false-negative results.

Our PCR-based assay, which targets the *iboH* gene associated with IBO/MUS biosynthesis, exhibits significant advantages over LC-MS. PCR instruments and reagents are approximately 100-fold cheaper than those for LC-MS, and the experimental workflow is simpler, making it more suitable for primary hospitals and centers for disease control (CDC). Although a single PCR run usually takes about 1 h (vs. usually 0.5 h per sample for LC-MS), PCR allows simultaneous processing of multiple samples, whereas LC-MS operates in a single-sample sequence. Moreover, DNA is more stable than IBO/MUS in fungal specimens [15,33], enabling PCR successful detection even after toxin degradation.

In the present study, we employed PCR-based detection for *iboH* as a molecular screening tool to assess the toxigenic potential of 53 representative *Amanita* species across eight sections of the genus. When we cross-referenced our PCR results with previously published LC-MS data and poisoning case reports presenting with IBO/MUS-like symptoms (Table 1 and Figure 5), we found that the concordance between PCR-based findings and chemical/clinical evidence reached 100%. The overall concordance between the presence of *iboH* and the presence of IBO/MUS supports the utility of our PCR-based assay for assessing toxigenic potential.

Our PCR-based assay also has inherent limitations that must be acknowledged. Most critically, PCR detects the presence of the *iboH* gene rather than the toxin itself, a positive result indicates toxigenic potential but does not confirm that toxins are actually being produced or accumulated in detectable quantities. This method provides no quantitative information about toxin concentration, which is often crucial for assessing poisoning severity.

Despite these limitations, the PCR-based assay established in this study offers a low-cost, rapid, and operationally simple alternative that is well-suited for primary hospitals in preliminary poisoning screening, as well as for local CDC laboratories in poisoning investigation and food risk assessment. In future work, the development of isothermal amplification techniques, such as loop-mediated isothermal amplification (LAMP) and rolling circle amplification (RCA), could further reduce the reliance on thermal cycling equipment, improve operational convenience, and enable accessibility for on-site-detection.

## Figures and Tables

**Table 1:** Species and specimens included in phylogenetic analysis and PCR assay, with examined specimens and newly generated sequences in this study in bold.

Section	Species	Voucher	ITS	LSU	*iboH*	IBO/MUS
Amanita	*A. albocreata*	RET 547-7	KU248128			✓ [37]
	*A. alpinicola*	MONT CLC2376	NR158316 [38]			
	*A. altipes*	HKAS 3660	AY436445 [39]			
	***A.** **altipes***	**MHHNU 33050**	C_AA120855 [33]	C_AA120982 [33]	**+, PZ793121**	✓ [15]
	*A.* ‘*amerlpanthera*’	RET 387-10	MT445432	HQ539707		
	*A. aprica*	RET 128-5	NR154683 [40]	NG057029 [40]		* [40]
	*A.* ‘*austrowellsii*’	RET 302-1	MN963578	MN963578		
	*A. bingensis*	KM 104	MT446264 [41]	MT446281 [41]		
	*A. breckonii*	NY 00066695	KJ535439	KJ535440		
	*A.* cf. *albopulverulenta*	KM 48		MT446282 [41]		
	*A. chrysoblema*	RET 320-1	EU071911 [42]	EU071984 [42]		✓ [43]
	***A.** **collariata***	**MHHNU 31095**	OM955206 [5]	OM955204 [5]	**+, PZ793122**	* [5,20]
	*A. concentrica*	CBM FB-24901	NR119387 [44]			✓ [15]
	*A. crenulata*	RET 675-9	MK204468	MK204474	+ [32]	✓ [45]
	*A. cruzii*	BARONI 8998	KC855222 [46]	KC855222 [46]		
	*A. diemii*	MES 1335		KY053387 [47]		E [48]
	*A. digitosa*	BBH 32154	KT213722 [49]			✓ [50]
	*A. elata*	HKAS 83449	MH508334 [1]	MH486486 [1]		
	***A.** **farinosa***	**MHHNU 32693**	ON131732 [15]	ON139027 [15]	**-**	× [15]
	*A. farinosa*	MHHNU 32768	ON131733 [15]	ON139028 [15]		
	*A. fibrillopes*	PERTH08793573	MN918103 [51]	MN918099 [51]		
	*A. flavoalba*	CAL 1405		KY861748 [52]		
	***A.** **flavomelleiceps***	**MHHNU 33119**	PQ326880	PQ330908	**+, PZ793123**	
	***A.** **flavopantherina***	**MHHNU 32267**	ON131735	ON139030	**+, PZ793124**	✓ [15]
	*A. flavopantherina*	HKAS 82613	MH508355 [1]	MH486519 [1]		
	*A. frostiana*	RET 543-6/RET 7-25-92 E	KP313580	AF024453 [53]		✓ [54]
	*A. gemmata*	JM96/62		AF097371 [55]		✓ [26]
	*A. gioiosa*	ML61952AG	MH603599 [56]			
	*A. gloeocystidiosa*	BBH 31903	KT213719 [49]			
	*A. griseopantherina*	HKAS 83560	MH508385 [1]	MH486573 [1]		
	***A.** **griseopantherina***	**MHHNU 33084**	C_AA120883 [33]	C_AA121009 [33]	**+, PZ793125**	✓ [15]
	*A.* ‘*hallingiana*’	RET 292-7	KY924833			
	*A. ibotengutake*	CBM FB-30969/30974	NR119388 [28]	AB088767 [28]		
	*A. kalasinensis*	CMUB-39966	OM040562 [4]	OM040553 [4]		
	*A. melleialba*	HKAS 83446	MH508430 [1]	KR824767 [1]		
	***A.** **melleialba***	**MHHNU 10606**	C_AA120884 [33]	C_AA121010 [33]	**+, PZ793126**	* [18]
	***A.** **melleiceps***	**MHHNU 31865**	ON131765 [15]	ON139065 [15]	**+, PZ793127**	* [19,20,21]
	*A. melleiceps*	MHHNU 32763	ON131767 [15]	ON139067 [15]		
	*A. minima*	KM 38	MT446261 [41]	MT446280 [41]		
	*A. mira*	HKAS 91953	MH508437 [1]	MH486646 [1]		* [57]
	*A. multisquamosa*	RET 374-1/BW RP14	MK840916 [58]	HQ539710		✓ [37]
	***A.** **muscaria***	**MHHNU 33653**	C_AA120885 [33]	C_AA121011 [33]	**+, PZ793128**	✓ [15,24]
	*A. muscaria*	MHHNU 31764	ON131738	ON139033		
	*A. nehuta*	JAC10720	MT863749	MT862256		
	*A. orientigemmata*	HKAS 80978	MH508469 [1]	MH486708 [1]		
	***A.** **orientigemmata***	**MHHNU 32700**	ON131764 [15]	ON139063 [15]	**+, PZ793129**	* [17,18,21]
	*A. pakistanica*	RET 317-6	KX365198	KX365199		
	*A. pantherina*	MB-102863	MH508488 [1]	MH486743 [1]	+ [32]	✓ [29]
	*A. pantherinoides*	iNAT 25463910	OM212868			✓ [59]
	*A. parcivolvata*	RET 614-4	MN963585	MN963584		
	*A. parvipantherina*	HKAS 83663	MH508499	MH486752		
	***A.** **parvipantherina***	**MHHNU 32942**	C_AA120859 [33]	C_AA120986 [33]	**+, PZ793130**	* [20,21]
	***A.** **parvisychnopyramis***	**MHHNU 32953**	PQ326876 [6]	PQ330907 [6]	**+, PZ793131**	✓ [6]
	*A. persicina*	RET 151-4	EU071892 [42]	EU071969 [42]		✓ [37]
	*A.* ‘*praecox*’	BW-PH082906-9		HQ539725		✓ [37]
	*A. pseudopantherina*	HKAS 80007	MH508514 [1]	MH486777 [1]		
	***A.** **pseudopantherina***	**MHHNU 11333**	C_AA120861 [33]	C_AA120988 [33]	**+, PZ793132**	✓ [15]
	*A. pseudosychnopyramis*	HKAS 87999	MH508530 [1]	KR824778 [1]		
	***A.** **pseudosychnopyramis***	**MHHNU 32762**	MZ313998 [15]	OM753110 [15]	**+, PZ793133**	✓ [15]
	*A. pudica*	RET 344-40		HQ539730		
	*A. pubescens*	RET 802-10	MH930991	MH931002		
	*A. pyriformis*	BBH 38643	KT213723 [49]			
	*A. ravicrocina*	CMUB 39967	OM040568 [4]	OM040559 [4]		
	*A. regalis*	HKAS 56699	MH508537 [1]	MH486797 [1]		✓ [31]
	*A. rubrovolvata*	MHHNU 32524	ON131751 [15]	ON139046 [15]		
	***A.** **rubrovolvata***	**MHHNU 32949**	C_AA120864 [33]	C_AA120991 [33]	**+, PZ793134**	✓ [15]
	*A. rufoferruginea*	HKAS 84974	MH508580 [1]	MH486843 [1]		
	***A.** **rufoferruginea***	**MHHNU 31100**	ON131773 [15]	ON139073 [15]	**+, PZ793135**	✓ [15]
	*A. robusta*	YA KM 41	NR173187 [41]	MT446278 [41]		
	*A. roseotincta*	RET 313-5	MH508551 [1]			
	*A. siamensis*	HKAS 67855	MH508592 [1]	MH486864 [1]		
	***A.** **siamensis***	**MHHNU 31578**	ON131776 [15]		**+, PZ793136**	* [20,21,60]
	*A. sinensis*	HKAS 80012	MH508597 [1]	MH486871 [1]		
	***A.** **sinensis***	**MHHNU 8585**	MK239263 [15]	ON139047 [15]	**+, PZ793137**	
	*A. stranella*	TENN 60935	FJ596814 [61]			
	*A. subfrostiana*	HKAS 32513		AF024477 [53]		
	***A.** **subfrostiana***	**MHHNU 32955**	C_AA120873 [33]	C_AA120999 [33]	**+, PZ793138**	
	*A. subglobosa*	HKAS 12009		AF024478 [53]		
	***A.** **subglobosa***	**MHHNU 32538**	ON131753 [15]	ON139051 [15]	**+, PZ793139**	✓ [15]
	*A. submelleialba*	CMUB S1	NR184934 [62]	NG088232 [62]		
	***A.** **subparcivolvata***	**MHHNU 32368**	OM955213 [5]	OM955211 [5]	**-**	× [15]
	*A. subparcivolvata*	MHHNU 32907	OM955216 [5]	OM955205 [5]		
	*A. subparvipantherina*	HKAS 56986		KR824776 [63]		
	***A.** **subparvipantherina***	**MHHNU 32226**	ON131756 [15]	ON139054 [15]	**+, PZ793140**	+
	*A.* ‘*subvelatipes*’	RET 483-10	MK204467			
	*A. sychnopyramis*	MHHNU 32153	ON131761 [15]	ON139060 [15]		
	***A.** **sychnopyramis***	**MHHNU 33330**	C_AA120879 [33]	C_AA121005 [33]	**+, PZ793141**	✓ [15]
	*A. taiepa*	JAC12826	MT863755	MT862263		
	*A.* ‘*tlaxcalipanthera*’	MO 428571	MW633035			
	*A. velatipes*	RET 489-2	MH508643 [1]	MH486938 [1]		
	*A. viscidolutea*	ANMF 767	MW000473			
	*A. wellsii*	RET 259-1	KU248116	OK285331		
	*A. xylinivolva*	ANDES F312 NVE56	FJ890024 [64]	FJ890036 [64]		
Amarrendia	*A. oleosa*	H7627	GQ925400 [65]	GQ925377 [65]		
	*A. umbrinella*	PSC 1813	AY194981	HQ539753		
Amidella	*A. rufobrunnescens*	GDGM 42374	KT865208 [66]	KT865210 [66]		
	***A.** **rufobrunnescens***	**MHHNU 33470**	**PZ832437**		**-**	
	*A. volvata*	S. Harsch 304		AF024485 [53]		
Arenariae	*A. arenaria*	VPI679		GQ925382 [65]		
Caesareae	*A. caesarea*	HKAS 96166	MH508283 [1]	MH486418 [1]		E [67]
	*A. kitamagotake*	HKAS 107309B	MW258874 [68]	MW258921 [68]		
	***A.** **kitamagotake***	**MHHNU 33459**	**PZ832438**	**PZ830472**	**-**	E [57]
	***A.** **princeps***	**MHHNU 31274**	PV564110 [69]	**PZ830473**	**-**	
	*A. princeps*	HKAS 122502	ON794332 [70]			
	*A. yuaniana*	HKAS 68662	MH508654 [1]	MH486957 [1]		
	***A.** **yuaniana***	**MHHNU 34553**	**PZ832439**	**PZ830474**	**-**	E [57]
Phalloideae	*A. exitialis*	HKAS 75775	JX998026 [71]	JX998053		
	***A.** **exitialis***	**MHHNU 20088**	**PZ832440**		**-**	
	*A. fuliginea*	MHHNU 9047	MN061271 [72]			
	***A.** **fuliginea***	**MHHNU 34221**	**PZ832441**		**-**	
	*A. pallidorosea*	MHHNU 8112	KU311697 [73]			
	***A.** **pallidorosea***	**MHHNU10523**	**PZ832442**	**PZ830475**	**-**	
	*A. phalloides*	HKAS 75773	JX998031 [71]	JX998060 [71]		
	*A. rimosa*	MHHNU 7954	KU311695 [74]			
	***A.** **rimosa***	**MHHNU 11109**	**PZ832443**	**PZ830476**	**-**	
	*A. subjunquillea*	BJTC FM2244	PV265320 [75]	PV259883 [75]		
	***A.** **subjunquillea***	**MHHNU 32832**		**PZ830477**	**-**	
	*A. subpallidorosea*	HKAS 85403	KP691683 [76]	KP691692 [76]		
	***A.** **subpallidorosea***	**MHHNU 34182**	**PZ832444**		**-**	
Roanokenses	*A. atrobrunnea*	MFLU 15-1415		NG057090 [49]		
	***A.** **atrobrunnea***	**MHHNU 34151**		PX699301	**-**	
	*A. caojizong*	HKAS 79673	MH508291 [1]	MH486429 [1]		
	***A.** **caojizong***	**MHHNU 32855**		**PZ830478**	**-**	E [57]
	*A. eijii*	HKAS 70229		MH486484 [1]		
	*A. eijii*	**MHHNU 10639**		**PZ830479**	**-**	
	***A.** **gymnopus***	**MHHNU 33197**		PX699306	**-**	
	*A. gymnopus*	HKAS 71618	MH508393 [1]	MH486582 [1]		
	*A. kotohiraensis*	MHHNU 6998	FJ176722 [77]	FJ011681 [77]		
	***A.** **kotohiraensis***	**MHHNU 33391**	PX696944	PX699307	**-**	
	*A. manginiana*	HKAS 101404		MH486637		
	***A.** **manginiana***	**MHHNU 34165**		PX699310 [1]	**-**	
	*A. oberwinklerana*	BJTC FM3352		PV259872 [74]		
	***A.** **oberwinklerana***	**MHHNU 34779**		**PZ830480**	**-**	
	*A. rubiginosa*	HKAS 52216	MH508561 [1]	MH486817 [1]		
	***A.** **rubiginosa***	**MHHNU 9701**		**PZ830481**	**-**	
Strobiliformes	*A. griseoverrucosa*	HKAS 38459	AY436486 [1]	AY436495 [1]		
	***A.** **griseoverrucosa***	**MHHNU 20089**		**PZ830482**	**-**	
	*A. strobiliformis*	MB-001177	MH508614 [1]	MH486895 [1]		
Vaginatae	*A. brunneofuliginea*	HKAS 83536	MH508268 [1]	MH486392 [1]		
	***A.** **brunneofuliginea***	**MHHNU 33664**	**PZ832445**		**-**	
	*A. changtuia*	HKAS 92100	NR159572 [1]	NG064562 [1]		
	***A.** **changtuia***	**MHHNU 20090**	**PZ832446**		**-**	
	*A. hamadae*	HKAS 79076	MH508394 [1]	MH486584 [1]		
	***A.** **hamadae***	**MHHNU 34767**	**PZ832447**		**-**	
	*A. umbrinolutea*	HKAS 89637	MH508638 [1]	MH486934 [1]		
	***A.** **umbrinolutea***	**MHHNU 34330**	**PZ832448**	**PZ830483**	**-**	
Validae	*A. brunneolimbata*	HKAS 78459	MH508274 [1]			
	***A.** **brunneolimbata***	**MHHNU 33436**	**PZ832449**	**PZ830484**	**-**	
	*A. excelsa*	HKAS 31510	AY436453 [39]	AY436491 [39]		
	*A. flavoconia*	HKAS 87990	MH508345 [1]	MH486508 [1]		
	***A.** **flavoconia***	**MHHNU 34414**	**PZ832450**	**PZ830485**	**-**	
	***A.** **pseudofritillaria***	**MHHNU 34731**	**PZ832451**	**PZ830486**	**-**	
	*A. pseudofritillaria*	MHKMU T. Huang 398	ON768708 [78]			
	*A. rubescens*	HKAS 100631		MH486810 [1]		
	***A.** **rubescens***	**MHHNU 34775**		**PZ830487**	**-**	E [79]
	*A. sepiacea*	HKAS 74862B	MW258870 [69]			
	***A.** **sepiacea***	**MHHNU 34722**	**PZ832452**	**PZ830488**	**-**	
	*A. sinocitrina*	HKAS 83445	MH508601 [1]	MH486876 [1]		
	***A.** **sinocitrina***	**MHHNU 34746**	**PZ832453**	**PZ830489**	**-**	× [80]
	*A. submaculata*	P19FSMF	MW589098 [81]			
	***A.** **submaculata***	**MHHNU 32842**	PV564112 [69]	**PZ830490**	**-**	
Lepidella	*A. flavofloccosa*	HKAS 90174/92006	MH508352 [1]	MH486516 [1]		
	*A. vittadinii*	HKAS 101430	MH508651 [1]	MH486950 [1]		

“+”, species confirmed to contain *iboH*; “-”, species confirmed to lack *iboH*; blank, no data or not confirmed; “✓”, species reported to contain IBO/MUS; “×”, species reported to lack IBO/MUS; “E”, species reported to be edible; “*”, species with documented human poisoning cases presenting symptoms consistent with IBO/MUS exposure.

## Data Availability

The DNA sequences obtained in this study have been submitted to GenBank.

## Supplementary Materials

The following supporting information can be downloaded at: https://www.mdpi.com/article/10.3390/jof12090691/s1, Supplementary Material S1: ITS-LSU sequence alignment.
